# Pumping infusions with a syringe may cause contamination of the fluid in the syringe

**DOI:** 10.1038/s41598-021-94740-1

**Published:** 2021-07-29

**Authors:** Yutaka Kawakami, Takashi Tagami

**Affiliations:** 1 Department of Critical Care Medicine, Odawara Municipal Hospital, Odawara, Japan; 2 Molecular Medical Bioscience Laboratory, Department of Medical Life Science, Yokohama City University Graduate School of Medical Life Science, Yokohama, Japan; 3 Department of Emergency and Critical Care Medicine, Nippon Medical School Musashikosugi Hospital, 1-396 Kosugimachi, Nakahara-ku, Kawasaki, Kanagawa 211-8533 Japan

**Keywords:** Medical research, Preclinical research, Translational research

## Abstract

Clinicians often perform pumping of infusions with a syringe (PIS) to quickly deliver fluid or blood transfusion to patients, especially during an emergency. Despite the efforts of the clinicians, critically ill patients are prone to acquire catheter-related bloodstream infections. Although clinicians have reported the possibility of PIS contamination, no group of researchers has studied nor confirmed this possibility. Here, we examined whether PIS can cause bacterial contamination of the fluid inside the syringes, using microbiological tests, including the analysis *Escherichia coli* DH-5 alpha growth by measuring the absorbance at OD_600_. We confirmed that contamination of fluid in the barrel was almost proportional to the applied volume of bacterial fluid. Aliquots of DH-5 alpha artificially applied on the surface of the gloved hand of an examiner, the plunger or the inner side of the barrel of a syringe could permeate inside the syringe. Furthermore, disinfection with ethanol before PIS almost successfully prevented bacterial multiplication. Our findings suggest that PIS can cause intraluminal contamination when performed with unsterilized hands, and that previous disinfection with ethanol can effectively prevent PIS-induced contamination. These results highlight the risk of PIS-induced contamination and the importance of disinfection in the daily clinical practice.

## Introduction

In the perioperative practice at an emergency department or intensive care unit, clinicians, such as emergency physicians, anesthesiologists, intensivists, and nurses, encounter critically ill patients requiring rapid diagnosis and resuscitation, such as those undergoing a cardiovascular surgery, or suffering from a hypovolemic shock caused by traumatic, massive, and acute gastrointestinal bleeding, or a septic shock caused by acute pan-peritonitis^1^. In order to treat these critically ill patients, healthcare providers often perform pumping infusions with a syringe (PIS) to intravenously and rapidly deliver a large volume of fluid or perform blood transfusions^2^. While performing PIS, a disposable sterile syringe should be carefully loaded at the lumen side of the intravenous line of the patient to avoid contamination of the solution by direct contact with the hands of the clinician or unsterilized gloves, according to the recommended good clinical practices^3,4^. However, when clinicians treat critically serious patients with refractory shock vital signs, appropriate aseptic maneuvers including hand washing or disinfection with ethanol tend to be left aside because of the emergency and shortage of time. Catheter-related blood stream infections (CRBSI) account for about twenty percent of total nosocomial bacterial infections^5^, which can substantially decline the perioperative health status of the patients^6–10^. In general, the four main causes of CRBSI are the presence of skin organisms, contaminated catheter hubs and infusates, and hematogenous infections from distant sites^11,12^. Although CRBSI mostly occurs in patients who received a central rather than a peripheral venous catheter, the latter can also cause CRBSI and related severe complications^13^. On the other hand, even though most clinicians believe that PIS itself constitutes a risk of contamination, no report has yet demonstrated that it can cause intraluminal contamination, or that patients who receive PIS during perioperative treatments are more likely to contract CRBSI than those who do not. A previous study showed that the use of a "blood conservation system," in which the syringe had a plastic cover that prevented its physical contact with the hand of the clinician during blood sampling, has lower chances of causing intraluminal contamination than that of a three-way stopcock catheter^14^; however, the precise mechanism of contamination was not elucidated. A few studies have shown PIS-induced contamination by hazardous drugs, such as chemotherapeutic agents^15,16^, but these reports mainly focused on the risk for health care providers, rather than on the risk of contaminating the reagents to be injected during syringe loading or patient administration. Taken together, PIS may be a risk factor for intraluminal contamination, but it had not been confirmed by microbiological investigations until now.

Here, we examined whether PIS can cause bacterial contamination of the fluid in a syringe barrel and trigger bacterial trans-catheter infections, by performing microbiological analyses of *E. coli* cultures. We found that artificially applied bacteria on a plunger, a syringe barrel, and on the surface of the hand of an examiner penetrated into the intraluminal fluid. Furthermore, we also found that disinfection with ethanol before PIS prevented bacterial contamination. These findings implicate that PIS itself can cause intraluminal contamination. Our data also indicate that disinfection is effective for prevention of bacterial contamination. These results reconfirm the importance of disinfection when PIS is required during perioperative, emergency, and intensive care clinical practice.

## Methods

### Bacterial cultures

To establish an assay system suitable for this study that could not be easily contaminated with other bacteria, we adopted an specific bacterial culture system. DH-5 alpha (*E. coli* DH-5 alpha Competent Cells, Takara Bio, Kusatsu City, Shiga, Japan) is a commercially available *E. coli* strain with high transformation efficiency for the incorporation of exogenous DNA that is frequently used for genetic manipulations in molecular cell biology research. DH-5 alpha was multiplicated in LB Miller medium (2.5%, Nacalai Tesque, Kyoto City, Kyoto, Japan); however, LB is not a selective medium for *E. coli* amplification.

### Pumping infusions with a syringe (PIS)

All of the procedures were performed in a safety cabinet to ensure the safety of the examiner and to prevent bacterial contamination from other sources. Briefly, the bacterial contamination of each of the following samples was determined (Fig. 2a): 2 mL of sterilized 2.5% LB Miller medium placed into the barrel of a new sterilized 10-mL plastic syringe (Terumo, Shibuya Ward, Tokyo, Japan) (negative control; NC), 10 µL of LB Miller medium containing DH-5 alpha 1–2 × 10^6^ cells/mL with 2 mL of sterilized LB Miller medium placed into the barrel of a new sterilized 10-mL plastic syringe (positive control; PC), 40 µL of LB Miller medium containing 1–2 × 10^6^ cells/mL of DH-5 alpha applied on the plunger with a micropipette (plunger contact; PL), 40 µL of LB Miller medium containing 1–2 × 10^6^ cells/mL of DH-5 alpha applied on the inner side of the barrel (IB), 2 mL of LB Miller medium placed into the barrel and LB Miller medium containing 1–2 × 10^6^ cells/mL of DH-5 alpha applied on sterilized gloves before PIS (contaminated gloves; GL), and 2 mL of LB Miller medium placed into the barrel and LB Miller medium containing 1–2 × 10^6^ cells/mL of DH-5 alpha, which were first applied on gloves, and then the gloves were sprayed with 80% ethanol and finally allowed to dry before PIS (disinfection of contaminated gloves with ethanol; GL + EtOH) (Fig. 2b). Also, we examined the effect of pulling the plunger down and up in different directions: the “vertical” method in which the plunger was pulled vertically, and the “inclined” method, in which the plunger was forcefully pushed down in an inclined direction with the tip of a syringe almost closed so that intraluminal LB Miller medium leaked through the gap between the edge of the plunger and a barrel (Fig. 3b) by originating a negative pressure in the barrel. Each syringe plunger was moved back and forth 10 times between the “3 and 12 mL” marks in the barrel, for 4 s per stroke, according to different methods of syringe usage (Fig. 2b). Then, each LB Miller medium sample in the barrel was transferred into a different sterilized test tube and cultured for about 19 h at 37 °C while shaking the solution (BR-43FL, Taitec, Nagoya City, Aichi, Japan).

### Measurement of OD_600_

Absorbances at 600 nm (OD_600_) of 96 µL aliquots per sample, for all the samples were measured with xMark Microplate Reader (Bio-Rad, Hercules, CA, USA). Each OD_600_ was calculated as follows:

$$ {\text{OD}}_{600} \;{\text{of}}\;{\text{each}}\;{\text{sample}} - {\text{OD}}_{600} \;{\text{of}}\;2.5\% \;{\text{LB}}\;{\text{Miller}}\;{\text{medium}} $$

### Statistical analysis

All the tests were performed in triplicate using Prism 7 software for Mac OS X (GraphPad software, San Diego, CA, USA). Statistical data are presented as mean ± standard error of the mean.

## Results

### Establishment of experimental PIS conditions without any other bacterial contamination

For this study, we set up different experimental conditions. DH-5 alpha, a highly competent *E. coli* strain, was grown in LB medium. To confirm the sterility of the growth medium, we performed two tests. For the first one, we soaked our fingers, covered with sterile nitrile gloves, into the medium; while for the second one, we immersed our bare fingers into the LB medium. Both media were incubated at 37 °C overnight and their OD_600_ values were measured. There are several methods to determine bacterial grow, among them colony forming unit and OD_600_ values has been widely used in research articles and guidelines on infections^3,4^. In our study, we have adopted OD_600_ values as bacterial grow indicator because is faster and easier to performed while also providing accurate results.

**Figure 1:**
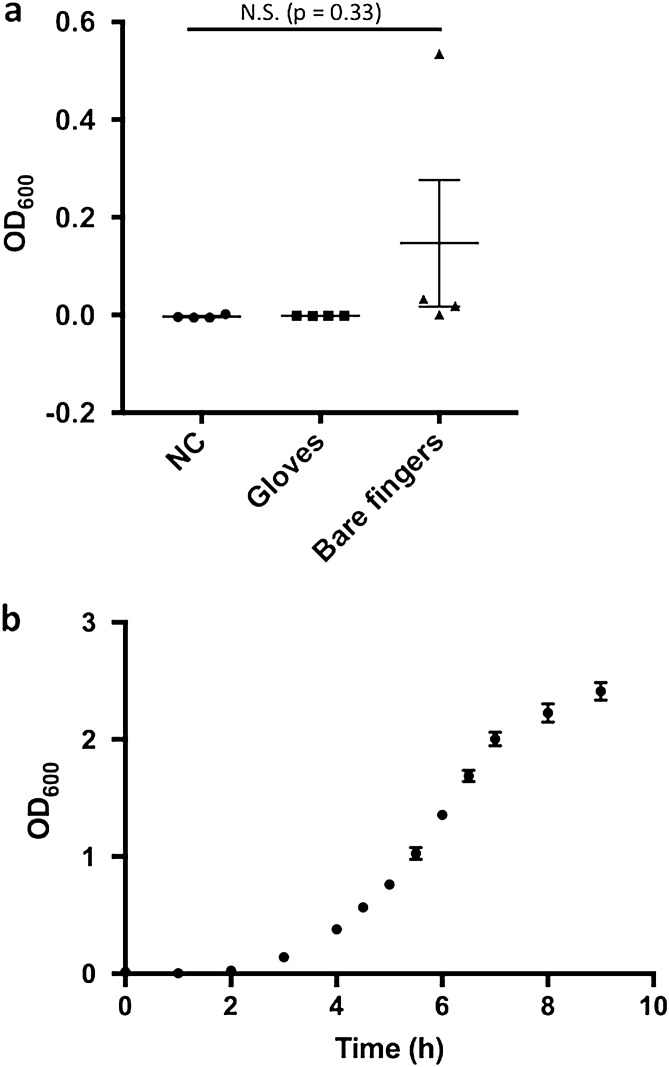
Experimental PIS conditions established. (**a**) NC (negative control) indicates no-treated medium. Values are expressed as mean ± SEM, n = 4; One-way ANOVA. (**b**) Growth curve of DH-5 alpha. At time 0, 10,000 cells/mL (2 mL) of DH-5 alpha were inoculated in LB medium and cultured in constant shaking conditions at 37 °C. Each indicator is shown as mean ± SEM, n = 4.

Insertion of sterile nitrile gloves did not result in any bacterial growth in the medium, as opposed to the use of bare fingers (Fig. 1a, *p* = 0.33). Based on this result, we performed all the remaining procedures with sterile gloves.

In order to confirm that the DH-5 alpha bacterial growth was almost proportional to the OD_600_ values, we cultured DH-5 alpha at a very low concentration (1 × 10^5^ cells/mL, OD_600_ = almost zero) and measured OD_600_ at sequentially consecutive time points. We observed that the OD_600_ values increased in time along with bacterial growth, between OD_600_ values of 0 and 2 (Fig. 1b). Based on these observations, we established as our experimental conditions those in which the OD_600_ values increased along with the number of DH-5 alpha, in the range of OD_600_ values between 0 and 2.

### PIS contaminated the fluid inside the loaded syringes

**Figure 2:**
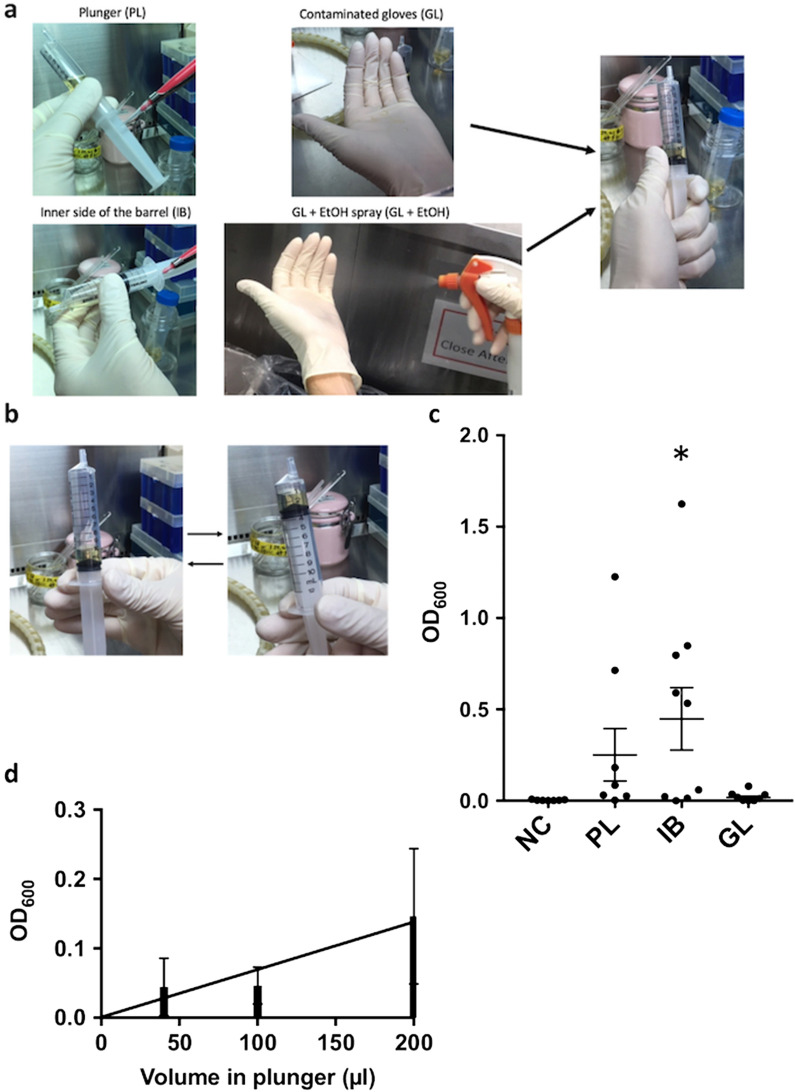
Pumping infusion with a syringe caused intraluminal contamination. (**a**) Representative images of each type of contamination and procedure. For the GL + EtOH condition, contaminated gloves were exposed to 80% ethanol spray and dried in the cabinet for a few minutes. (**b**) Each PIS was performed as follows: a plunger was moved between “3 and 12 mL” marks in one stroke and 2 s, 10 strokes were performed in total, and then the intraluminal medium present in the syringe was transferred into a sterile test tube. (**c**) Comparison between different types of contamination in PIS. Values are expressed as mean ± SEM, n = 9, *p < 0.05, ANOVA post-hoc Dunnett's multiple comparison test. (**d**) Intraluminal contamination in a plastic syringe. Each volume of DH-5 alpha-containing medium was applied on a plunger, and PIS was performed. The absorbance values almost proportionally increased and fitted to the Y = 0.0007 × X + 0.0009 equation, with R^2^ = 0.9254. Values are expressed as mean ± SEM, n = 4.

To determine whether PIS could induce bacterial contamination, we tested several scenarios that could lead to contamination: 40 µL of DH-5 alpha droplets were applied at the plunger (PL), 40 µL were applied with a micropipette inside the barrel around the “10 mL” indicator (IB), 200 µL were applied on top of gloved hands (contaminated gloves; GL, Fig. 2a). For PL and IB conditions, we performed ten strokes between the “3 and 12 mL” indicators for about 4 s per stroke by grasping the edge of the plunger (Fig. 2b). For GL and GL + EtOH conditions, we grasped the whole plunger with the treated gloves during the strokes (Fig. 2a). We determined that PL, GL, and in particular IB conditions caused bacterial contamination (Fig. 2c). In addition, to examine whether the degree of PIS-induced contamination in the intraluminal medium was dependent on the number of strokes after adding the aliquot of bacteria in the syringe, these procedures were repeated with several volumes of DH-5 alpha-containing medium. This assay determined that the bacterial contamination was almost proportional to the applied volume (R^2^ = 0.9254, Fig. 2d). These results suggested that PIS that contained bacteria in the plunger, lumen, and hand of the examiner caused bacterial contamination in an originally aseptic intraluminal fluid.

### Disinfection with ethanol before PIS prevented PIS-induced contamination

**Figure 3:**
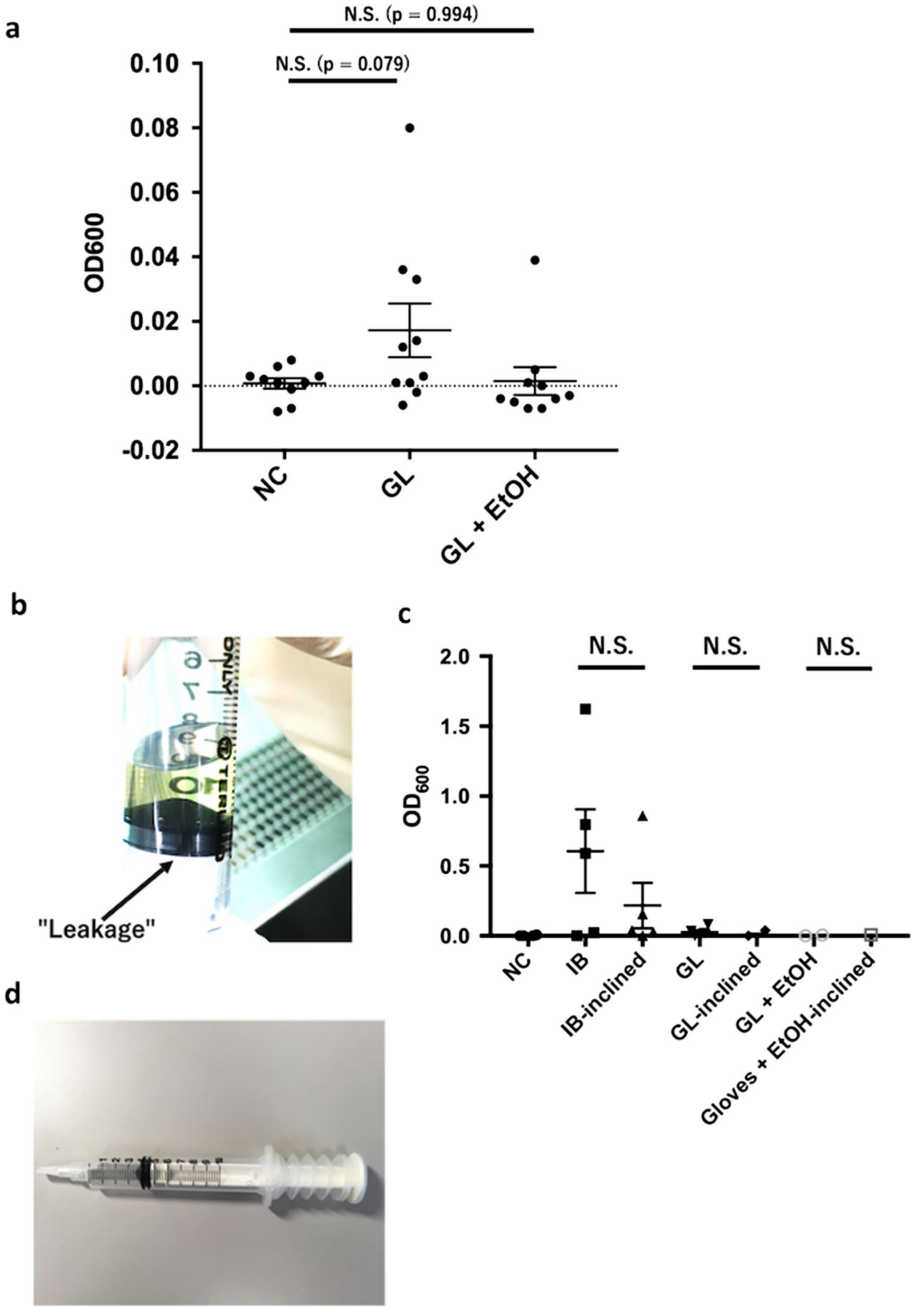
Disinfection with ethanol before PIS prevented PIS-induced contamination. (**a**) Comparison between NC, GL, and GL + EtOH conditions. Values are expressed as mean ± SEM, n = 10, ANOVA post-hoc Dunnett's multiple comparison test. (**b**, **c**) Investigation on whether leakage of LB Miller medium through a lumen and a plunger induced intraluminal contamination. Values are expressed as a mean ± SEM, n = 5, Student's t test. (**d**) Example of a commercially available plastic syringe with a plastic cover that prevents direct physical contact of the hand of the clinician with the plunger (Edwards Lifesciences, Irvine, CA, USA).

Next, to determine whether disinfection by spraying the syringe with ethanol before PIS prevented bacterial contamination, we compared both GL and GL + EtOH conditions (200 µL of DH-5 alpha-containing medium applied on sterile gloved hands followed by 80% of ethanol spray, Fig. 2a). We noted that addition of EtOH prevented further, although not statistically significant, GL-induced contamination, compared to GL conditions, (Fig. 3a, *p* = 0.074, ANOVA post-hoc Dunnett's multiple comparison test).

Finally, clinicians often experience the leakage of intraluminal reagent into the rubber edge of a plunger during PIS, especially when PIS is performed quickly (Fig. 3b) and the plunger is rapidly and forcefully pulled at an inclined angle, because the negative pressure produced inside the barrel of a syringe can easily draw air through the gap between the plunger and the barrel. We hypothesized that this leakage could introduce bacteria attached around the plunger into the syringe and thus cause intraluminal contamination. To examine this, we performed strokes at a normal angle (vertical) and at an inclined angle with leakage at the edge of a plunger (inclined). We observed that the inclined procedure did not result in increased intraluminal contamination (Fig. 3c). Taken together, a simple and easy disinfection of the syringe with ethanol before PIS was able to reduce the risk of intraluminal contamination; however, the precise mechanism of bacterial contamination is still unknown.

## Discussion

In this study, we examined whether the artificial application of bacterial culture on a syringe plunger or lumen, or the glove of the examiner can cause the penetration of the bacteria into the fluid inside the syringe, using a microbiological methodology. We demonstrated for the first time that PIS itself could cause trans-catheter bacterial contamination by any of the conditions examined in this study. Furthermore, we also confirmed that previous disinfection with ethanol could prevent PIS-induced bacterial contamination.

Several previous studies have highlighted the risk of contamination in clinical practice from different aspects^17–20^. Stucky et al. emphasized the influence of pollutants in the clinical environment on the risk of contamination inside a syringe^19^. In the present experiments, we showed that PIS caused intraluminal contamination, although the contamination induced by considerably contaminated gloves was not significantly increased compared to loading bacterial droplets on the plunger or the inner side of the barrel. We also showed that previous disinfection of the syringe by spraying ethanol almost prevented the contamination. Therefore, these results provided a very important take-home lesson for all clinicians. During the perioperative period and critical care, where PIS is often performed for patient resuscitation, the incidence of CRBSI is one of the highest risk factors for a longer intensive care unit stay, that could lead to devastating complications, such as refractory severe sepsis, infectious endocarditis, and death^7,21^. Therefore, to minimize the possible risk of contamination all health care providers are required to apply good clinical practices as hygienically as possible. As this study demonstrated, previous disinfection is a very simple and easy procedure that would be beneficial for the prevention of iatrogenic complications.

Our study also presented scientific validity data on the role of the plastic cover outside the plunger of the syringe on preventing contamination by preventing physical contact of the plunger with external materials, shown in Fig. 3d. Closed-type peripheral arterial catheters, which are commonly used in intensive care unit and operation room for real-time blood pressure monitoring and easy blood access, have adopted this type of covered plastic syringes, but no study has been reported on its efficiency to prevent contamination. Our study shows that the plastic cover would be effective in the prevention of intraluminal contamination. Further experiments using the covered disposable plastic syringe may enable us to better understand the mechanism of contamination of the fluid in the barrel.

We observed a relatively large variability between the results of our experimental replicates (Fig. 2c). The reason is unknown; however, when we tested if the way of moving the plunger could affect the degree of contamination, no significant differences were obtained (Fig. 3c). It could be possible that a very small amount of DH-5 alpha-containing medium was pulled into the gap between the plunger and the barrel and that the resulting contamination depended on the amount of trapped volume. The mechanism of this phenomenon is still unknown, but one conceivable hypothesis is that the syringes used in our study were plastic disposable ones, and they might have been deformed by the slight heat produced during forced and repeated strokes, resulting in a small gap sufficient for penetration of bacteria into the intraluminal medium. To verify this hypothesis, further investigation on PIS-induced contamination, such as using glass-made or heat-resistant plastic syringes, is required.

Our study had some limitations. First, not all of the PIS procedures, in which a plunger was moved for 4 s per stroke, were perfectly performed at the same speed, pressure, and friction force between the plunger and barrel. The absorbance values obtained in each experiment, which greatly differed in number, indicated that a slight difference in the pressure and friction might deform the plastic lumen, resulting in very small bacterial penetration. Second, our results do not propose a strict warning that PIS with bare hands would cause bacterial contamination of loaded fluid or reagents in the syringe, and could lead to CRBSI with high possibility, because our study was just a simple experiment. Although several samples showed high absorbance in our experiment, they were considerably smaller than that of positive control (data not shown). This implies that the number of bacteria that penetrated the barrel might be very low, and these penetrations might be negligible in clinical practice. Finally, it is yet unknown whether PIS-induced contamination itself can actually cause CRBSI in clinical medicine. In this sense, several related factor such as the detailed mechanism and growth pattern of PIS-induced contamination, the amount of bacteria outside the syringe or in the fluid inside the barrel that is sufficient to cause infection, the extent of the penetrated bacteria increase, and the extent to which the immunologic conditions of the patient receiving PIS get compromised for infection, remain unknown. Thus, future investigations on the detail of exacerbation of intraluminal contamination induced by PIS, possibly into infectious complications in animal models, such as rodents, are required.

In conclusion, we showed for the first time that PIS itself caused intraluminal contamination when bacteria are attached outside the barrel of a syringe, and that previous disinfection of gloves prevented bacterial contamination. Our results confirmed the importance of keeping the hands of the clinicians antiseptic during management of the catheter of a patient, according to the recommended good clinical practice, and performing a disinfection prior and during surgery, emergency medical treatments, and intensive care to protect the health of the patient.

## Data Availability

All data generated or analyzed during this study are included in this published article.
